# Breast Cancer After Treatment of Differentiated Thyroid Cancer With Radioiodine in Young Females: What We Know and How to Investigate Open Questions. Review of the Literature and Results of a Multi-Registry Survey

**DOI:** 10.3389/fendo.2020.00381

**Published:** 2020-07-10

**Authors:** Christoph Reiners, Rita Schneider, Tamara Platonova, Mikhail Fridman, Uwe Malzahn, Uwe Mäder, Alexis Vrachimis, Tatiana Bogdanova, Jolanta Krajewska, Rossella Elisei, Fernanda Vaisman, Jasna Mihailovic, Gracinda Costa, Valentina Drozd

**Affiliations:** ^1^University Hospital, Würzburg, Germany; ^2^The International Fund “Help for Patients With Radiation-Induced Thyroid Cancer ‘ARNICA”’, Minsk, Belarus; ^3^University Hospital, Münster, Germany; ^4^Institute of Endocrinology and Metabolism, Kiev, Ukraine; ^5^M. Sklodowska-Curie National Research Institute of Oncology, Gliwice, Poland; ^6^University Hospital, Pisa, Italy; ^7^National Cancer Institute, Rio de Janeiro, Brazil; ^8^Institute of Oncology Voijvodina, Sremska Kamenica, Serbia; ^9^University Hospital, Coimbra, Portugal

**Keywords:** differentiated thyroid carcinoma, radioiodine therapy, iodine-131, long-term complications, young females, childhood and adolescence, second primary malignancy, breast cancer

## Abstract

Published studies on the risk of radiation-induced second primary malignancy (SPM) after radioiodine treatment (RAI) of differentiated thyroid cancer (DTC) refer mainly to patients treated as middle-aged or older adults and are not easily generalizable to those treated at a younger age. Here we review available literature on the risk of breast cancer as an SPM after RAI of DTC with a focus on females undergoing such treatment in childhood, adolescence, or young adulthood. Additionally, we report the results of a preliminary international survey of patient registries from academic tertiary referral centers specializing in pediatric DTC. The survey sought to evaluate the availability of sufficient patient data for a potential international multicenter observational case–control study of females with DTC given RAI at an early age. Our literature review identified a bi-directional association of DTC and breast cancer. The general breast cancer risk in adult DTC survivors is low, ~2%, slightly higher in females than in males, but presumably lower, not higher, in those diagnosed as children or adolescents than in those diagnosed at older ages. RAI presumably does not substantially influence breast cancer risk after DTC. However, data from patients given RAI at young ages are sparse and insufficient to make definitive conclusions regarding age dependence of the risk of breast cancer as a SPM after RAI of DTC. The preliminary analysis of data from 10 thyroid cancer registries worldwide, including altogether 6,449 patients given RAI for DTC and 1,116 controls, i.e., patients not given RAI, did not show a significant increase of breast cancer incidence after RAI. However, the numbers of cases and controls were insufficient to draw statistically reliable conclusions, and the proportion of those receiving RAI at the earliest ages was too low.In conclusion, a potential international multicenter study of female patients undergoing RAI of DTC as children, adolescents, or young adults, with a sufficient sample size, is feasible. However, breast cancer screening of a larger cohort of DTC patients is not unproblematic for ethical reasons, due to the likely, at most slightly, increased risk of breast cancer post-RAI and the expected ~10% false-positivity rate which potentially produced substantial “misdiagnosis.”

## Introduction

Treatment of differentiated thyroid cancer (DTC) in childhood, adolescence, or early adulthood with surgery, radioiodine (iodine-131, I-131) therapy (RAI), and thyroid hormone replacement achieves 10-year survival rates of 95%, with relatively low recurrence rates of 10–30% (1). However, an excellent long-term survival may be partly offset by an increased risk for second primary malignancy (SPM) related to RAI or other causes.

According to a systematic review by Clement et al. (2), the risk for SPM is increased after RAI of DTC. For many years, the gastrointestinal tract (salivary glands, stomach, and colorectum), the genitourinary tract (kidneys and bladder), and the hematopoetic system (blood cells) have been considered to be at risk to develop SPM after RAI (2–4). However, a recent meta-analysis did not find an increased risk of solid cancers after RAI (5).

Since I-131 is concentrated by the sodium iodide symporter and that molecule is expressed in the mammary gland (6), the female breast may receive relevant radiation doses between 0.2 and 2 Gy from repeated courses, with cumulative activities of 1–15 GBq (7). Based on the recently introduced radiation risk assessment tool of the United States National Cancer Institute, a dose of 2 Gy to the breast of a 10-year-old girl hypothetically doubles her lifetime risk for breast cancer, whereas in a 50-year-old woman, the risk increases only by 20% (8).

## Objective and Scope

Published studies on the risk of radiation-induced SPM, including breast cancer in DTC, refer mainly to middle-aged and older adult survivors. These studies are not easily generalizable to those treated as children, adolescents, or young adults since the patients who are still growing are more sensitive to radiation, and the patients treated earlier in life may have a longer potential latency period. Therefore, we here (1) review available literature on the risk of breast cancer as an SPM (A) independent of the type of treatment of RAI and (B) after RAI of DTC, with a focus on young females, and (2) report the results of our preliminary international multi-registry survey to evaluate the availability of patient data for a sufficiently powered multicenter international study of DTC patients given RAI as children, adolescents, or young adults. This research project was sponsored by the German Federal Office for Radiation Protection.

## Review of the Literature

### Methods

The literature included in the analysis was identified by two independent reviewers (VD and RS), principally using an automated literature search for English language papers published from 1984 to 2018 regarding breast cancer after RAI of DTC. The search was carried out using the online Medline (PubMed), Cochrane, and Embase databases. The main search terms were “thyroid” OR “breast” OR “mamma” AND “cancer” OR “malignancy” OR “carcinoma” OR “tumor” AND “second primary malignancy” AND “radioiodine therapy.” Besides the automated search, a manual search for additional relevant publications was made of the bibliographies of the papers identified automatically.

Systematic reviews, meta-analyses, cohort studies, and case–control studies were included. Case reports, single-center studies with small databases or study samples (*N* < 500), narrative reviews of the literature, editorials, and letters to the editor were excluded. Also excluded were publications regarding studies not differentiating between synchronous and metachronous SPMs.

From the publications fulfilling the inclusion criteria, the following information (if available) were extracted:

- First author, date, country
- Setting (e.g., hospital registry and population-based registry), single-center or multicenter design
- Duration of the study and of the follow-up time
- Study sample characteristics (number, gender, and age of patients/controls), exclusion criteria in the study
- DTC characteristics (histology and TNM stage)
- Surgery, RAI yes/no, cumulative I-131 activity as a surrogate for radiation dose
- SPM (of all kinds/breast cancer) before/after RAI
- Latency period, age dependency, dose dependency of SPM, and breast cancer incidence (correlation with I-131 activity)
- Risk estimates for secondary breast cancer after DTC (independent of treatment) and for secondary breast cancer after RAI of DTC, expressed as one or more of the following:
  ◦ absolute number
  ◦ excess absolute risk
  ◦ excess relative risk
  ◦ observed/expected incidence (O/E)
  ◦ odds ratio
  ◦ relative risk
  ◦ standardized incidence ratio (SIR)
  ◦ incidence rate ratio
  ◦ hazard ratio (HR)

with corresponding 95% confidence interval (CI).

Because of the heterogeneity and the variable design of the studies in the literature, only a qualitative and not a pooled analysis was performed.

### Results

#### Publications Included

**Figure 1:**
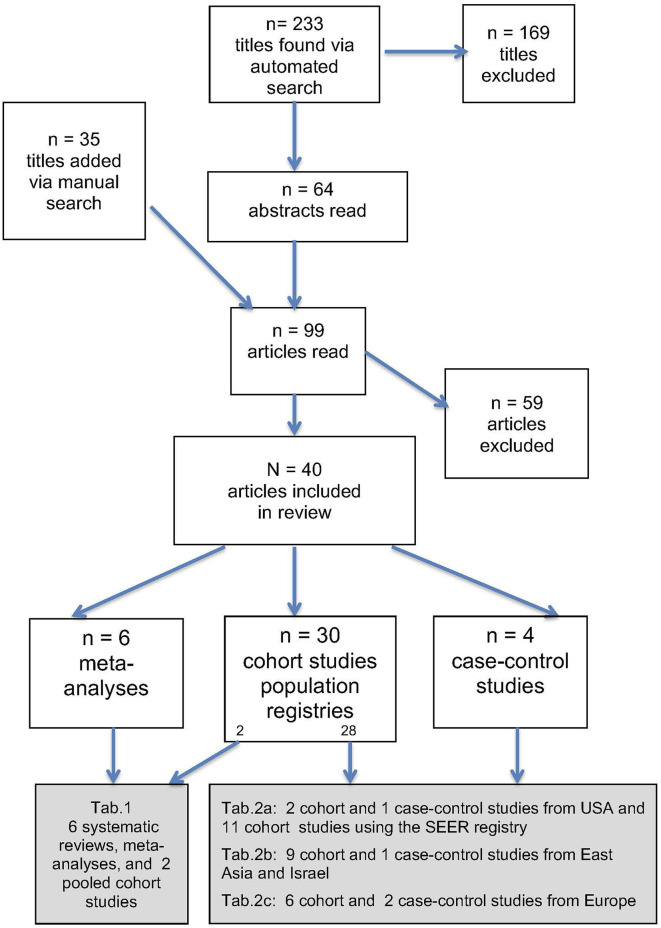
Flow diagram describing the literature search process.

Using the search strategy described above, a total of 233 citations were found (Figure 1), 169 of which were excluded because the publications were unrelated to DTC, represented duplicate publication of the same studies, or were otherwise deemed not to be relevant. In addition to the 64 publications located through the automated search that were deemed to be of potential interest based on their abstracts, a manual search considering the bibliographies of the review articles retrieved 35 citations deemed to be of potential interest based on their abstracts. Thus, 99 full-text articles were read, of which 59 were excluded for the following reasons: small number of clinical observations by single centers (*n* = 12), case reports (*n* = 11), letters to the editor (*n* = 4), and unclear specification if the breast cancer was diagnosed before or after DTC (*n* = 32) (the papers could be excluded on more than one grounds).

Thus, altogether 40 publications were included in our analysis (Figure 1); they are cited and summarized in four tables. The main criterion for the assignment of a publication to a particular table was the type of study: Table 1—systematic reviews and meta-analyses and Table 2—cohort or case–control studies. Since the majority of the studies analyzed were large single-center cohort or case–control studies, resulting in a large number of publications, such articles were assigned to “sub-tables” according to the region of origin (Table 2A—USA, Table 2B—East Asia and Israel, and Table 2C—Europe). Within the tables, the studies are arranged according (1) to country of origin and (2) year of publication (chronological order from earliest to most recent).

**Table 1:** Breast cancer risk in DTC survivors and/or DTC survivors given RAI: systematic reviews, meta-analyses, and internationally pooled cohort studies.

**References, country**	**Study design, setting**	**Study period, length of FU (y), exclusion if FU < x(y), lost to FU (%)**	**Age range or mean age at DTC diagno-sis (y)**	**DTC cases (TR), DTC with RAI (TR)**	**BC cases (BT), BC with RAI (BR)**	**BC risk after DTC, BC risk after RAI. Risks (95%Ci)**	**BC risk status after DTC:**	**BC risk status after RAI:**
Subramanian et al. (9), USA, Canada	Systematic review of the literature and meta-analysis, 8 pooled studies	1966–2006, 6–15 y		TC 60,490		SIR(BT) = 1.25 (1.17–1.32)	**⇑**	
Sawka et al. (10), Canada	Systematic review of the literature and meta-analysis, 2 pooled studies	1966–2008, 9–13 y, 1 y excluded		TC 16,502, TR 8,473		RR(BR) = 0.86 (0.64–1.16)		⇔
Joseph et al. (11), Australien	Systematic review of the literature and meta-analysis, 18 pooled studies	1946–2015, <2 y excluded		TC 223,782		SIR(BT) = 1.24(1.16–1.33)	**⇑**	
Zhang et al. (12), China	Systematic review of the literature and meta-analysis, 6 pooled studies	1934–2009, 7.8–12 y	42–50 y	TC 17,914, TR 9,000	BR 96	RR(BR) = 061 (0.47–0.79)		⇔
Nielsen et al. (13), USA	Systematic review of the literature and meta-analysis, 18 pooled studies	1934–2009, 7.8–12 y		TC 44,879	BT 5,791	OR(BT) = 1.18 (1.09–1.26)	**⇑**	
Yu et al. (5), Canada	Systematic review of the literature and meta-analysis, 7 pooled studies	2008–2017, 7–13 y		TC 68,481	BT 1,276	RR(BR) = 0.8 (0.53–1.21)	⇔	
Rubino et al. (14), France	Pooled 3-cohort study, French, Swedish, Italian cohorts	1934–1995, 13 y, <2 y excluded	44 y	TC 6,841	BT 128, BR 54	SIR(BT) = 1.3 (1.0–1.5), SIR(BR) = 1.2 (0.9–1.6), RR = 0.8 (0.5–1.1)	**⇑**	⇔
Sandeep et al. (15), Europe, Canada, Australia, Singapore	Pooled cohort study 13 cancer registries of Europe, Canada Australia, Singapore	1953-2000, 25 y, <1 y excluded		TC 39,002	BT 552	SIR(BT) = 1.31 (1.21–1.43)	**⇑**	

*Some data may be absent in particular rows if the data were not reported in the publication. Up arrows (⇑) denote increased risk, horizontal arrows (⇔) denote no increased risk. Unless otherwise noted, the “DTC patients” group includes both patients receiving RAI and those not receiving RAI*.

*BC, breast cancer; CI, confidence interval; DTC, differentiated thyroid cancer; FU, follow-up; OR, odds ratio; RAI, radioiodine therapy; RR, relative risk; SIR, standardized incidence ratio*.

**Table 2A:** Breast cancer risk in DTC survivors and/or DTC survivors given RAI: cohort and case-control studies from USA.

**References, country**	**Study design, setting**	**Study period, length of FU (y), exclusion if FU < x (y), lost to FU (%)**	**Age range or mean age at DTC diagnosis (y)**	**DTC cases (TC), DTC with RAI (TR)**	**BC cases (BT), BC with RAI (BR)**	**BC risk after DTC (BT), BC risk after RAI (BR), Risks (95% CI)**	**BC risk status After DTC**	**BC risk status after RAI**
Vassilopoulou-Sellin et al. (16), USA	Cohort study, University of Texas and SEER registry	1944–1997, <2 y	42 y	TC 1,013	BT 24, BR 14	All ages RR(BT) = 3.9 (0.5–28.6), 40–49 y RR(BT) = 3.0 (1.17–8.62)	⇔	⇑
Chen et al. (17), USA	Cohort study SEER Registry	1973–1994 <2 y	48.6 y	TC 23,080	BT 252	RR(BT) = 3.9 (1.04–1.33)	**⇑**	
Ronckers et al. (18), USA	Cohort study SEER Registry	1973–2000, 8 y, <2 mo	43 y	TC 29,456	BT 530, BR 53	O/E(BT) = 1.21 (1.11–1.32), O/E(BR) = 1.18 vs. O/E (no BR) = 1.28	**⇑**	⇔
Bhattacharyya et al. (19), USA	Cohort study SEER Registry	1988–2001, RAI 5.2 y, no RAI 4.7 y,	RAI 43.5 y, no RAI 54 y	TC 29,231, TR 10,349	BT 424, BR 112	Prevalence of BR 1.08% of BT without RAI 1.6%,		⇔
Chuang et al. (20), USA	Cohort study SEER Registry	1973–2000, RAI 15 y no RAI 11.1 y, <6 mo	>18 y	TC 26,639	BT 462, BR 344	RR(BT) = 1.02 (0.81–1.29), RR(BR) = 0.86 (0.6–1.24)	⇔	⇔
Brown et al. (3), USA	Cohort study SEER Registry	1973–2002, 8.6 y	42 y	TC 30,278	BT 597, BR 76	All ages O/E(BT) = 1.22 (1.12–1.32), <25y O/E(BT) = 1.16 (0.58–2.08) All ages O/E(BR) = 1.21 (0.95–1.52)	**⇑**	⇔
Kim et al. (21), USA	Cohort study SEER Registry	1973–2008		TC 52,103	BT 1,041	SIR(BT) = 1.13 (1.06–1.20), SIR(BR) = 1.14 (0.98–1.31)	**⇑**	⇔
Kuo et al. (22), USA	Cohort study, SEER Registry	1990–2011	46 y	TC 38,158, TR 16,670	BT 954, BR 384	OR(BT) = 1.02 (1.01–1.02), OR(BR) = 0.94 (0.82–1.08)	**⇑**	⇔
Uprety et al. (23), USA	Cohort study SEER Registry	2004–2010, <6 mo, 12.8 y	>18 y	TC 12,603	BT 291	O/E(BT) = 1.19 (1.06–1.34)	**⇑**	
Endo et al. (24), USA	Cohort study, SEER Registry	1992–2013, <6 mo	61 y	TC 75,992	BT 727, BR 245	O/E(BT) = 1.17 (1.09–1.26), O/E (BR) = 1.08 (0.95–4.7), O/E(no BR) = 1.12 (1.01–1.24)	**⇑**	⇔
Adly et al. (25), USA	Cohort study, SEER Registry	1973–2013	16 y	TC 1,769	BT 9	SIR (BT) = 0.96 (0.44–1.83)	⇔	
Ron et al. (26), USA	Cohort study, Connecticut Tumor Registry	1935–1978, <2, mo1 6%	47.3 y	TC 1,618, TR 281	BT 34, BR 8	SIR(BT) = 1.89 (1.31–2.64), SIR(BR) = 2.57 (1.1–5.07)	**⇑**	**⇑**
Simon et al. (27), USA	Case-control study National Institute of Child Health and Human	1961–1995	35–64 y	TC 23	BT 4,575	OR(BT) = 2.7 (1.2–5.9)	**⇑**	
Canchola et al. (28), USA	Cohort study, California Cancer Registry	1988–1999, <1 y		TC 10,932	BT invasive 78, BT *in situ* 23	SIR (invasive) = 0.9 (0.7–1.1), SIR (*in situ*) = 1.6 (1.0–2.4)	⇔ ⇑	

*Some data may be absent in particular rows if the data were not reported in the publication. Up arrows (⇑) denote increased risk, horizontal arrows ⇔ denote no increased risk. Unless otherwise noted, the “DTC patients” group includes both patients receiving RAI and those not receiving RAI*.

*BC, breast cancer; CI, confidence interval; DTC, differentiated thyroid cancer; FU, follow-up; O/E, observed/expected ratio; OR, odds ratio; RAI, radioiodine therapy; RR, relative risk; SIR, standardized incidence ratio*.

**Table 2B:** Breast cancer risk in DTC survivors and/or DTC survivors given RAI: cohort and case-control studies from Asia.

**References, country**	**Study design, setting**	**Study period, length of FU (y), excluded if FU < x(y), lost to FU (%)**	**Age range or mean age at DTC diagnosis (y)**	**DTC cases (TC), DTC with RAI (TR)**	**BC cases (BT), BC with RAI (BR)**	**BC risk after TC, BC risk after RAI, Risks (95% CI)**	**BC risk status after DTC**	**BC risk status after RAI**
Cho et al. (29), Korea	Cohort study, Korean Central Cancer Registry	1993–2010, <2 mo excluded	45.2 y	TC 178,844	BT 599	SIR(BT) = 1.20 (1.11–1.30)	**⇑**	
Ahn et al. (30), Korea	Cohort study, Registry of Seoul National University Hospital	1973–2012, 5 y, <2 y excluded	45.2 y	TC 6,150, TR 3,631	BT 99	HR(BR) = 0.49 (0.22–1.06)		⇔
Ahn et al. (30), Korea	Case-control study, Registry of Seoul National University Hospital	1970–2009, 5 y, <2	43.4 y	TC 4,243	BT 55	SIR(BT) = 2.45 (1.83–2.96)	**⇑**	
Khang et al. (31), Korea	Cohort study, Registry of Seoul National University Hospital	1976–2010, 7 y, <1 y excluded	46.4 y	TC 2,468, TR 1,396	BT 17	BT was more frequent, in the no RAI group.	**⇑**	
Lu et al. (32), Taiwan	Cohort study, Taiwan Cancer	1979–2006, 7.1 y, <1 mo excluded	45.2 y	TC 19,068	BT 102	SIR(BT) = 1.42 (1.16–1.72)	**⇑**	
Teng et al. (33), Taiwan	Cohort study, Taiwan National Health Insurance Database	1997–2010, 6.5 y, <1 y	46 y	TC 20,235, TR 11,799	BT 158	SIR(BT) = 1.48 (1.26–1.73), HR(BR) = 0.99 (0.96–1.02)	**⇑**	⇔
Lin et al. (34), Taiwan	Cohort study, Taiwan National Health Insurance Database	2000–2011, 5.9 y	46 y	TC 10.361, TR 7,069	BT 129, BR 91	HR(BT) = 1.31(1.07–1.61), HR(BR) = 1.34(1.06–1.69)	**⇑**	**⇑**No correlation with activity
Sadetzki et al. (35), Israel	Cohort study, Israel National Cancer Registry	1960–1998, 9.4 y, <1 y excluded		TC 4,911	BT 70	SIR(BT) = 1.07 (0.84-1.34)	⇔	
Hirsch et al. (36), Israel	Cohort study, Israel National Cancer Registry, Rabin Medical Center Thyroid CancerRegistry	9.3 y, <2 y excluded	48.1 y	TC 1,943, TR 1,574	BT 49, BR 39	The most common SPM was breast cancer(49 from 173)	**⇑**	
Izkhakov et al. (37) Israel	Cohort study, Israel National Cancer Registry	1980–2011, 9.7 y, <1 y excluded	51.2 y (Jews), 41.4 y (Arabs)	TC 11,538	BT 258	SIR(BT) = 1.44(1.26-1.61)	**⇑**	

*Some data may be absent in particular rows if the data were not reported in the publication. Numbers in parentheses represent 95% CI's. Up arrows (⇑) denote increased risk, horizontal arrows (⇔) denote no increased risk. Unless otherwise noted, the “DTC patients” group includes both patients receiving RAI and those not receiving RAI*.

*BC, breast cancer; CI, confidence interval; DTC, differentiated thyroid cancer; FU, follow-up; HR, hazard ratio; RAI, radioiodine therapy; SIR, standardized incidence ratio*.

**Table 2C:** Breast cancer risk in DTC survivors and/or DTC survivors given RAI: cohort and case-control studies from Europe.

**References, country**	**Study design, setting**	**Study period, length of FU (y), excluded if FU < x(y), Lost to FU (%)**	**Age range or mean age at DTC diagnosis (y)**	**DTC cases (TC), TC with RAI (TR)**	**BC cases (BT), BC with RAI (BR)**	**BC risk after TC, BC risk after RAI, risks (95% CI)**	**BC risk status after DTC**	**BC risk status after RAI**
Osterlind et al. (38), Denmark	Cohort study, Denmark Cancer	1943−1980, 5.9 y		TC 1,351	BT 11	SIR(BT) = 0.96 (0.76–1.20)	⇔	
Hall et al. (39), Sweden	Cohort study, Swedish Cancer	1958–1975, <1 y excluded	49 y	TC 2,968	BT 45	SIR(BT) = 0.99 (0.72–1.33)	⇔	
Hall et al. (40), Sweden	Cohort study, Registry of 6 university hospitals	1950–1975, 14–16 y, <2 y excluded	5–75 y	TC 1,955, TR 834	BT 36, BR 9	SIR(BT) = 1.37 (0.91–2.00) FU > 10 y SIR(BT) = 1.75 (1.06–2.74), SIR(BR) = 0.74 (0.34–1.40),	⇔ ⇑	⇔
Hall et al. (41), Sweden	Case-control study, oncologic centers of 6 hospitals	1950–1975, 50 y	5–75 y	TC 1,955, TR 834	BR 36, no BR 107	O/R(BR) = 0.47 (0.21–1.08)		⇔
Akslen and Glattre (42), Norway	Cohort study, Cancer Registry of Norway	1955–1985, 8.8 y, <2 mo excluded		TC 2,720	BT 33	SIR(BT) = 1.03 (0.71–1.44)	⇔	
Adjadj et al. (43), France	Case-control study, 3 French cancer centers	1934–1995, 12 y, <2 y excluded, 21% lost to FU, 15% died	42 y	TC 2.365	BT 48	SIR(BT) = 1.3 (1.0–1.7), SIR(BR) = 1.2 (0.2–6.2)	**⇑**	⇔
Berthe et al. (44), France	Cohort study, Basse-Normandie Cohort	1960–1998, <1 y excluded	47 y	TC 875	BT 12	SIR(BT) = 1.19 (0.62–2.08)	⇔	
Edhemovic et al. (45), Slovenia	Cohort study, Cancer Registry of Slovenia	1971–1993, 5.2 y, <1 Mo excluded	54.9 y	TC 894	BT 4	SIR(BT) = 1.12 (0.31–2.87)	⇔	

*Some data may be absent in particular rows if the data were not reported in the publication. Numbers in parentheses represent 95% CIs. Up arrows (⇑) denote increased risk, horizontal arrows (⇔) denote no increased risk. Unless otherwise noted, the “DTC patients” group includes both patients receiving RAI and those not receiving RAI*.

*BC, breast cancer; CI, confidence interval; DTC, differentiated thyroid cancer; FU, follow-up; HR, hazard ratio; OR, odds ratio; RAI, radioiodine therapy; SIR, standardized incidence ratio*.

Specifically, Table 1 is a list of the results of six systematic reviews and meta-analyses. In addition, two large multinational pooled cohort studies are shown.

The majority of the included published studies (*n* = 28) were cohort studies of large population-based tumor registries (Tables 2A–2C). The US Surveillance, Epidemiology, and End Results (SEER) Registry (Table 2A) served as the database for 11 publications. Besides the studies using the SEER registry, Table 2A presents three other American analyses: one single-center cohort study, one cohort study analyzing the SEER database plus a local registry, and one case–control study.

Seven cohort studies (Table 2B) from East Asia also used large registries, e.g., the databases of the Korean Central Cancer Registry, the Taiwanese National Health Insurance, and the Taiwanese National Cancer Registry. Three cohort studies (Table 2B) analyzed the Israel National Cancer Registry. From Europe (Table 2C), six large cohort studies were found.

Finally, four case–control studies (Tables 2B, 2C) were included in our review, the largest of which included more than 4,000 cases (46).

A considerable proportion of the articles tried to take the patients' age at the diagnosis of DTC into consideration. However, only two studies focused on the risk of breast cancer after RAI for DTC in children and adolescents (3, 25).

It should be mentioned that, of 40 studies, 22 dealt with the general risk of breast cancer after DTC, not taking into account the form of treatment. Fourteen of the 40 studies dealt both with the general risk and the RAI-dependent risk. Conversely, four studies considered only the risk of breast cancer after treatment of DTC with RAI.

#### Risk of Breast Cancer After DTC (Independent of Therapy)

Three large meta-analyses and two large pooled cohort studies (Table 1) revealed a generally increased risk for breast cancer as an SPM in DTC patients, too, with significant SIRs of about 1.2 (9, 11, 13–15). Interestingly, only one of the meta-analyses gave an indication to exclude such an effect (5).

Eight of the 12 cohort studies examining the SEER registry and other registries from the USA (Table 2A) also described significant, if weak, increases of breast cancer risk in DTC survivors, with SIRs of ca. 1.2 or O/E values of ca. 1.2 (3, 17, 18, 21–24, 26).

On the other hand, three studies on the SEER registry (16, 20, 25) and two other cohort studies from the USA (25, 28) did not reveal such increases of breast cancer risk in DTC survivors (Table 2A).

Slightly higher, and again significant, increases of breast cancer risk in patients after a diagnosis of DTC have been described by nine of 17 cohort and case–control studies from East Asia, Israel, and Europe (Tables 2B, 2C) (29, 31–34, 36, 37, 43, 46). The SIRs in these studies ranged between 1.3 and 1.5, with the exception of 2.5 in patients from a Korean registry (Table 2B) (46).

On the contrary, seven of the same 17 studies (Tables 2B, 2C), including one from Israel (35), four from Scandinavia (38–40, 42) as well as two from France and Slovenia (44, 45), did not show an increased general risk for breast cancer in DTC patients. Hall et al. (40) did find such an association, but only in their subgroup at follow-up of >10 years (Table 2C).

Studies looking at age as a risk modifier and comparing subgroups of adult patients suggested no clear age dependency of SIRs (Table 2A) (3, 16, 25). Brown et al. (3) compared the risk of breast cancer in two groups of relatively young DTC survivors, those younger than 25 years of age and those 25–49 years of age, respectively (Table 2A). The authors only found a significantly increased risk for breast cancer in the older group. A similar observation was made by Vassilopoulou-Sellin et al. (16), who found an increased risk for breast cancer in women ages 40–49 years, but not in younger patients (Table 2A). Most interestingly for our survey regarding age-related risks, the only study specifically addressing young patients, an analysis of the SEER database performed by Adly et al. (25), did not find an increased risk for breast cancer in patients younger than age 20 years at the time of DTC diagnosis and who were followed up for up to 40 years (Table 2A).

Few studies have investigated the latency times between diagnosis of DTC and detection of breast cancer. The above-mentioned study of the Korean National University Hospital, Seoul, database with the highest SIR, 2.5 (Table 2B) (46), reported in patients older than 30 years a mean latency time between diagnoses of DTC and breast cancer of 6.6 (minimum–maximum, 3.3–7.8) years. In patients diagnosed with DTC when younger than age 30 years, the mean latency time was considerably longer, 17.9 (minimum–maximum, 13.9–20.4) years.

#### Studies Comparing Breast Cancer Risk in Patients With DTC Receiving vs. Not Receiving RAI

The first cohort study describing an increased risk for breast cancer after DTC, that of Ron et al. (26), already focused on radiation as a risk factor for the former malignancy and reported a significantly increased SIR of 2.6 after radiation therapy (Table 2A). However, in the study of Ron et al. (26), the group of patients with radiation treatment was small (*n* = 8) and the type of treatment (percutaneous radiation therapy, RAI) was not specified.

On the contrary, two meta-analyses and one large pooled cohort study (Table 1) comparing the risk of breast cancer as an SPM in DTC patients given or not given RAI did not find an increased risk after RAI (10, 12, 14).

The absence of an association between RAI of DTC and breast cancer was confirmed by all seven studies on the risk of breast cancer after such treatment upon examining the US SEER Registry (Table 2A) (3, 18–22, 24). The same conclusions were drawn in five of six studies in other countries, e.g., Korea and Taiwan (Table 2B) (30, 33), the Scandinavian nations (40, 41), and France (Table 2C) (43).

In detail, Ahn et al. (30) noted that the risk of breast cancer was not associated with RAI (Table 2B); even for relatively high cumulative activities of ≥4.4 GBq, no effect was demonstrated. Lin et al. (34) showed that there was a small increase of breast cancer risk in DTC patients post-RAI (Table 2B), but not as high as that associated with DTC *per se*. Most importantly, in patients receiving cumulative activities of >4.4 GBq in comparison to patients receiving <4.4 GBq, the risk for breast cancer was not increased.

Brown et al. (3) did not find a correlation with RAI in any age group or when comparing breast cancer risk in two subgroups of relatively young patients after DTC—those <25 years old vs. those 25–49 years of age, respectively (Table 2A). Of interest is that Vassilopoulou-Sellin et al. (16) found, independent of RAI, an increased risk for breast cancer in women aged 40–49, but not in younger patients (Table 2A).

Adly et al. (25) also analyzed the SEER Registry data but focused on young patients (Table 2A). These investigators concluded that the overall risk of all kinds of SPM was significantly increased (SIR 1.5; 95%CI, 1.08–1.98) in patients undergoing RAI for DTC, being higher in females and White patients. Additionally, the cumulative incidence of all kinds of SPM after RAI of DTC in children appeared to increase steadily with survival after the primary treatment. The overall risk of SPM in patients with RAI was found to be significantly higher than expected compared to the risk in the general population. Based on this study, the pediatric thyroid cancer survivors are at an increased risk of developing SPM of the organs highly exposed to radiation by RAI: salivary glands, gums, and other parts of the mouth, the stomach, as well as the kidneys. By contrast, a significant increase of breast cancer as SPM in patients given RAI as children was not observed (SIR 0.96; 95%CI, 0.44–1.83).

## International Multi-registry Survey

### Participating Registries and Study Design

The second part of this report addresses a survey of patient registries from institutions known by the authors to specialize in treating children and adolescents with DTC. This preliminary study evaluated the availability of sufficient patient data to conduct an adequately powered international multicenter observational case–control study regarding breast cancer risk in female DTC survivors who were treated with RAI at a young age. Altogether eight academic tertiary referral centers from Germany, Ukraine, Poland, Italy, Brazil, Serbia, and Portugal agreed to participate. In addition, the German-Belarusian Foundation “ARNICA” contributed two separate datasets, one from their registry and the other from a dedicated smaller database which was set up for a “pilot study” that sought to test the feasibility of a multicenter international study on breast cancer risk in DTC survivors (Drozd et al., submitted). The nine institutions participating in the registry survey were:

- Foundation “ARNICA,” Minsk, Belarus (feasibility study sample and routine registry)
- Institute of Endocrinology and Metabolism, Kiev, Ukraine
- Instituto Nacional do Cancer—INCA, Rio de Janeiro, Brazil
- Institute of Oncology Vojvodina, Department of Nuclear Medicine, Sremska Kamenica, Serbia
- Department of Nuclear Medicine, University Hospital, Coimbra, Portugal
- Department of Nuclear Medicine, University Hospital, Münster, Germany
- Department of Endocrinology, University Hospital, Pisa, Italy
- Clinic of Nuclear Medicine and Endocrine Tumors, M. Sklodowska-Curie Memorial Cancer Center and Institute of Oncology, Gliwice, Poland
- Department of Nuclear Medicine, University Hospital, Würzburg, Germany

In the registry survey, in 2014–2015, the participating centers answered questionnaires requesting the most relevant information regarding breast cancer in female survivors of DTC, particularly those receiving RAI at an early age. These queries included the database's total number of female DTC survivors diagnosed during childhood or adolescence with or without a history of RAI, the percentage of DTC survivors who were ages <18 or <40 years of age at the time of the primary treatment, the cumulative activity of I-131 administered for RAI, and the follow-up regimen after RAI. Queries were also made regarding the incidence of SPM, specifically breast cancer, both before and after the DTC diagnosis. The registry study was performed with anonymous, aggregated patient data.

The pilot observational cohort study alluded to above was performed in collaboration with the Foundation “ARNICA” and other organizations in Belarus and Germany in 2016–2017 (Drozd et al., submitted). In this feasibility study, selected female patients given RAI for their DTC in childhood or adolescence and control patients not given RAI underwent clinical, imaging, and laboratory examinations as part of a screening program for breast cancer. As noted previously, the dedicated database that was created for the pilot study also was analyzed in the present study. To avoid duplicate publication, any patients in the dedicated database who also were in the Foundation “ARNICA” Registry were excluded from all analyses of the latter.

### Number and Origin of Patients and Controls

Tables 3A, 3B show the key aggregated data for each of the nine centers and 10 databases included in our survey. The cohorts comprised altogether 7,565 female DTC survivors given RAI (“RAI patients,” *n* = 6,449) or not given RAI (“controls”, *n* = 1,116). The number of RAI patients per institution varied considerably from 15 to 1,644 and the number of controls from 0 to 419.

**Table 3A:** Registry survey: RAI patient and control cohort characteristics by center - young age groups.

**Study Center**	**Feasibility study sample, ARNICA**		**Registry, ARNICA**		**Institute of Endocrinology & Metabolism**		**National Tumor-Institute (INCA)**		**Dept. Nuclear Medicine, Institute of Oncology Vojvodina**		**Dept. Nuclear Medicine, University Hospital**	
**City, country**	**Minsk, Belarus**		**Minsk, Belarus**		**Kiev, Ukraine**		**Rio de Janeiro, Brazil**		**Sremska Kamenica, Serbia**		**Coimbra, Portugal**	
***N*** (% of combined study sample)	202 (2.7%)		1,328 (17.6%)		1,297 (17.1%)		89 (1.2%)		32 (0.4%)		15 (0.2%)	
**Cohorts**	RAI patients	Controls^*^	RAI patients	Controls	RAI patients	Controls	RAI patients	Controls	RAI patients	Controls	RAI patients	Controls
***n*** (% of RAI patient or control cohort)	102 (1.6%)	100 (9.0%)	909 (14.1%)	419 (37.5%)	1,199 (18.6%)	98 (8.8%)	81 (1.3%)	8 (0.7%)	32 (10.5%)	0	15 (0.2)	0
**Current age** (years, M ± SD)	30.1 ± 1.9	34.3 ± 5.3	28.5 ± 5.2	36.7 ± 5.9		36.7 ± 5.9	25.6 ± 9.6	31.2 ± 8.9	29.3 ± 6.1		21.5 ± 5.1	
**Age at first surgery** (years, M ± SD)	11.9 ± 3.2	17.6 ± 6.6	13.3 ± 3.3	23.6 ± 9.1	29.6 ± 8.1	26.2 ± 7.6	14.50 ± 3.0	14.5 ± 3.3	15.8 ± 3.9		14.2 ± 2.6	
<18 years, *n* (%)				134 (32%)	190 (15.8%)	134 (32%)					15 (100%)	
<40 years, *n* (%)				416 (99.3%)	1,125 (93.8%)	416 (99.3%)						
**Age at first RAI** (years, M ± SD)	12.8 ± 3.0		14.3 ± 3.4				14.5 ± 3.0		16.9 ± 0.81		15.5 ± 2.0	
<18 years, *n* (%)									17 (53%)		15 (100%)	
<40 years, *n* (%)									15 (47%)			
**Cumul. I-131 activity** (GBq, M ± SD)	11.8 ± 9.5		6.6 ± 3.8				10.4 ± 5.5		10.9 ± 0.2		7.2 ± 8.0	
**Follow-up duration** (years, M ± SD)	17.3 ± 3.2	15.9 ± 3.4	17.4 ± 2.8	13.6 ± 6.2			11.1 ± 8.4	16.2 ± 8.2	13.1 ± 7.4		5.4 ± 4.5	
**SPM before DTC**												
Breast cancer, *n* (%)	0	0	0	0			0	0	0		0	
Other cancers, *n* (%)	0	0	8 (0.9%)	5 (1.2%)					0		0	
**SPM after DTC/RAI**												
Breast cancer, *n* (%)			1 (0.9%)	0			0	0	0		0	
Other cancers, *n* (%)			3 (0.3%)	1 (0.2%)					0		0	

*Cumul., cumulative; Dept., department; DTC, differentiated thyroid carcinoma; follic., I = 131, iodine-131; M ± SD, mean + standard deviation; RAI, radioiodine therapy; SPM, second primary malignancy*.

* *Controls were patients with DTC who had not received RAI*.

**Table 3B:** Registry survey: RAI patient and control cohort characteristics by center - all age groups.

**Study center**	**Dept. Nuclear Medicine, University Hospital**		**Dept. Endocrinology, University Hospital**		**Dept.Nuclear Medicine & Endocrinology, MSC Memorial Cancer Center**		**Dept.Nuclear Medicine University Hoispital**	
**City/country**	**Münster, Germany**		**Pisa, Italiy**		**Gliwice, Poland**		**Würzburg, Germany**	
***N*** (% of combined study sample)	1,808 (23.9%)		1,091 (14.4%)		867 (11.5%)		836 (11.1%)	
**Cohorts**	RAI patients	Controls^*^	RAI patients	Controls	RAI patients	Controls	RAI patients	Controls
***n*** (% of RAI patient or control cohort)	1,644 (25.5%)	164 (14.7%)	1,091 (16.9%)	0	650 (10.1%)	217 (19.4%)	726 (11.3%)	110 (9.9%)
**Current age** (years, M ± SD)	56.3 ± 16.2	55.5 ± 13.3						
**Age at first surgery** (years, M ± SD)	47.6 ± 15.9	48.8 ± 13.1	44.13 ± 3.0		43.3 ± 4.9	43.9 ± 2.1	45.6 ± 16.1	46.7 ± 15.5
<18 years, *n* (%)					44 (6.8%)	7 (3.2%)		
<40 years, *n* (%)					241 (37.1%)	76 (35.0%)		
**Age at first RAI** (years, M ± SD)	47.8 ± 15.9						45.7 ± 16.1	
<18 years, *n* (%)	34 (2.1%)						30 (4.1%)	
<40 years, *n* (%)	544 (33.1%)						244 (33.6%)	
**Cumul. I-131 activity** (GBq, M ± SD)	7.8 ± 10.1		6.0 ± 6.5		4.0 ± 5.8		7.5 ± 6.6	
**Follow-up duration** (years, M ± SD)	8.6 ± 6.8	6.8 ± 6.1	16.1 ± 10.4		11.4 ± 3.9	10.9 ± 4.3	4.7 ± 3.72	5.74 ± 4.08
**SPM before DTC**								
Breast cancer, *n* (%)	10 (0.6%)	3 (1.8%)	5 (0.5%)		4 (0.6%)	0	5 (0.7%)	1 (0.9%)
Other cancers, *n* (%)	20 (1.2%)	1 (0.6%)	9 (0.8%)		10 (1.5%)	0	107(14.7%)	5 (4.5%)
**SPM after DTC/RAI**								
Breast cancer, *n* (%)	16 (1.0%)	2 (1.2%)	27 (2.5%)		11 (1.6%)	0	9 (1.2%)	2 (1.8%)
Other cancers, *n* (%)	13 (0.8%)	4 (2.4%)	42 (3.8%)		14 (2.2%)	2 (0.9%)	19 (2.6%)	4 (3.6%)

*Cumul., cumulative; Dept., department; DTC, differentiated thyroid carcinoma; I = 131, iodine-131; M ± SD, mean + standard deviation; RAI, radioiodine therapy; SPM, second primary malignancy*.

* *Controls were patients with DTC who had not received RAI*.

The three largest groups of RAI patients, with more than 1,000 cases each, were contributed by the participating centers in Münster, Kiev, and Pisa. By contrast, by far the largest “no RAI” control group was provided by the Foundation “ARNICA,” Minsk, with a total of more than 500 controls (pilot study database plus registry). Table 3A is firstly a list of the three cohorts from Minsk and Kiev, which included a considerable proportion of young patients who developed DTC after the Chernobyl reactor accident in 1986 (1, 47).

### Cohort Characteristics

It is apparent from Tables 3A, 3B that not all information was provided by all centers. However, the data supplied seemed to suffice to answer the survey's most important questions about the number and the main characteristics of the patients/survivors under observation.

#### Age Distribution

The data in Tables 3A, 3B are listed according to the patients' and the controls' ages at the time of the first treatment (surgery and/or RAI). Table 3A shows the young patient groups from five centers, who were between 12 and 16 years of age at the time of their initial treatment. Only the patients and the controls from Kiev were older, with mean ages of 26–30 years. However, because the Kiev cohorts included 16% of patients and 32% of controls not older than 18 years, we present their data in Table 3A, too, directly adjacent to the data from Minsk.

The cohorts from the four other centers (Table 3B), which together contributed approximately two-thirds of the RAI patients (4,110/6,449), mirrored the typical age distribution for patients with DTC, with mean ages at surgery/first RAI between 43 and 48 years.

#### Cumulative I-131 Activity

Except for the cohort from Kiev, all centers reported their cumulative activity of I-131 (in GBq) administered for RAI (Tables 3A, 3B). The mean cumulative activities of the centers in Minsk (registry), Würzburg, Münster, and Coimbra ranged between 6.0 and 10.0 GBq, whereas the highest mean cumulative activities, >10–12 GBq, were applied in patients from Minsk (pilot study sample) as well as in Rio de Janeiro and Sremska Kamenica. The relatively high cumulative activities can be explained by the young age of the patient cohorts from Belarus, Brazil, and Serbia. That demographic characteristic presumably corresponded to a relatively aggressive disease and hence to high rates of nodal or distant metastases or both, requiring a greater I-131 activity to be effectively treated.

#### Duration of Follow-Up

Eight of the nine centers (Tables 3A, 3B) provided information about the mean duration of follow-up after RAI in RAI patients and after surgery in controls (when available). On average, the centers in Minsk and Pisa had the longest mean follow-up times, slightly over 15 years. The mean follow-up times of the centers in Gliwice, Rio de Janeiro, and Sremska Kamenica ranged between 11 and 13 years. The shortest follow-up times were reported from Münster, with about 9 years, and Würzburg and Coimbra, with about 5 years.

#### Second Primary Malignancies

Again, eight of the nine centers provided data about SPM and breast cancer before and after the diagnosis of DTC (Tables 3A, 3B). There was considerable inhomogeneity of the data reported, which can be explained by the age distribution of the different cohorts. In the very young cohorts from Minsk (pilot study sample), Rio de Janeiro, Sremska Kamenica, and Coimbra, no breast cancer cases were reported in patients or controls. The relatively large cohorts of patients (*n* = 909) and controls (*n* = 419) of the Minsk registry included a low number of breast cancer cases, eight and five, respectively, before the diagnosis of DTC. After the DTC diagnosis, there was one additional case of breast cancer and three additional cases of other SPM in the RAI patient group as compared to no case of breast cancer and one case of another SPM in the control group.

The rates of SPM and breast cancer were higher in the much older cohorts from Würzburg, Münster, Gliwice, and Pisa. The rates before and after the diagnosis of DTC reported by the latter three centers were very similar, ranging between 0.5 and 2.5% (median, 0.9%) for breast cancer and 0.6–4.5% (median, 1.9%) for SPM other than breast cancer. The exceptionally high rate of other malignancies before DTC in the Würzburg cohort may be explained, at least partially, by detection bias due to the existence of a comprehensive local clinical cancer registry and a follow-up program. In that cohort, systematic differences in the cumulative incidences of breast cancer and other SPM between RAI patients and controls were not recognizable.

## Discussion

### Literature Review

#### Breast Cancer Risk in Differentiated Thyroid Cancer Patients Generally Increased, Influence of RAI Questionable

In a comprehensive systematic review and meta-analysis, Nielsen et al. (13) investigated the relationship between breast cancer and DTC. Interestingly, these authors, in line with earlier investigators (17, 48), described a bi-directional association, meaning that the risk of breast cancer was increased in patients with DTC and *vice versa*.

The majority of studies addressing this issue that are referenced here (22/34) indicate that there is a generally increased risk for breast cancer after diagnosis of DTC, independent of therapy. This observation was reflected in five of six systematic reviews, meta-analyses, and pooled studies (Table 1). The observation also was echoed in eight of 12 cohort studies using the SEER registry or other registries from the USA (Table 2A) and nine of 16 studies analyzing other registries (Tables 2B, 2C).

In the general population of the USA, breast cancer risk in women below age 45 years corresponds to approximately one case in 87, or 1.2% (49). To give a rough estimate of the risk in DTC survivors, a SIR of 1.5, the maximum value for 90% of the studies listed in Table 1, would mean that, with the diagnosis of DTC, the general risk of 1.2% could be increased by 50%, to ~1.8%, in women younger than 45 years of age.

However, the only study specifically focusing on children and adolescents younger than 20 years old does not suggest an increased breast cancer risk in patients with DTC (25). There is some indication that the latency times for breast cancer after DTC in young patients are much longer than in adults, often lasting 20 years or more, so that the studies examining this risk have to focus on long observation times (30).

Beyond the generally increased risk for breast cancer in DTC survivors independent of treatment, a history of RAI seems not to have any additional influence on this risk (Tables 1, 2A, 2C), based on published findings of all SEER studies, all meta-analyses, and nearly all cohort studies from a variety of countries. There are two exceptions describing an increased risk in cohorts after RAI, but correlations of the degree of risk with the therapeutic activity of I-131 were not found (26, 34). Two additional studies examined the possibility of a higher risk of breast cancer in young patients given RAI, which was not confirmed (3, 16).

Assessing the hypothesis of a generally increased risk for SPMs other than breast cancer in DTC patients/survivors was not an objective of the present study. However, in young (as well as adult) DTC patients after RAI, a generally increased risk for SPM seems to exist, specifically in organs and tissues relatively highly irradiated by RAI, e.g., salivary glands, gums, and other parts of the mouth, the stomach, and the kidneys (25).

#### Limitations and Weaknesses of Published Studies

Concerning the risk of breast cancer in DTC survivors given RAI at a young age—the original focus of this review—data are inconclusive for many reasons. A general problem of critically reviewing publications from, e.g., tumor registries is that non-independent data sets from identical registries are analyzed and published repeatedly. This plays an important role in the context of our review because 11 of 40 studies utilized the SEER registry and, similarly, three studies from Korea, two studies from Taiwan, three studies from Israel, and two studies from Sweden all refer to the same respective databases from those countries (Tables 2A, 2C). As expected, the systematic reviews and the meta-analyses used these same databases, too. To give an example, the six such studies cited here (Table 1) in five cases include patients from Hall et al. (39–41), in four cases include those of Rubino et al. (14), and in three cases each were the samples of Adjadj et al. (43), Berthe et al. (44), and Brown et al. (3).

Generally, the risk for breast cancer in DTC patients independent of therapy may be overestimated since the samples in 36 studies on the general risk of breast cancer in DTC patients included a large proportion of patients who were treated with RAI. Therefore, the studies' estimates of breast cancer risk in “all DTC survivors” might be conservatively high—because any increased risk due to RAI would materially elevate risk in the overall group.

Detection bias is possibly the most relevant limitation for most of the studies published since patients with a cancer diagnosis tend to participate more strictly in cancer screening programs, e.g., for breast cancer. Detection bias, by the way, may be the cause for the surprisingly high rate, 14.7%, of SPM other than breast cancer before the diagnosis of DTC in the Würzburg registry. Another limitation of the literature is that the published studies often did not differentiate between synchronous and metachronous SPMs; indeed that is one reason why more than 30 papers were excluded from this literature review.

In addition, when comparing DTC patients after RAI with DTC controls without a history of that treatment, selection may have introduced a relevant bias. That is because the indication for RAI depends on tumor stage, and RAI usually is not performed in patients with the frequently favorable stage pT1N0M0 (50). Conversely, patients treated with RAI tend to suffer from more advanced DTC, and this propensity for more aggressive malignancy may also be reflected in a predisposition for SPM such as breast cancer.

Only one case–control study (28) investigated the risk of breast carcinoma *in situ* vs. invasive breast cancer in DTC patients, separately describing such an increased risk for carcinoma *in situ* only (Table 2A).

Latency times between radiation exposure and manifestation of solid cancers tend to require a minimum of 4–5 years, with the consequence that SPM presenting earlier probably is not radiation-induced. In some studies that we evaluated, the patients were excluded if the SPM appeared within a month, several months, or even longer periods of up to 2 years after the diagnosis of DTC—which may be still too short an exclusion threshold.

On the other hand, maximum latency times for radiation-induced SPM may reach up to 30–40 years after treatment. The studies analyzed here had mostly relatively short mean observation times of <10 years (*n* = 12 studies; median, 7 years) and less often long follow-up times of 10–20 years (*n* = 7 studies, median 12 years). According to the American Thyroid Association's “Management Guidelines for Children with Thyroid Nodules and Differentiated Thyroid Cancer,” the minimum follow-up time for studies on outcomes and long-term side effects of DTC therapy in children and adolescents should be at least 10 years (50).

A severe drawback in the context of potentially RAI-induced breast cancer in DTC patients is that data about the therapeutic activity of I-131 (in mCi or GBq) are mostly not reported. Moreover, this activity is merely a surrogate for the radiation dose to breast tissue (in Gy), which only can be determined by individual measurements of uptake and effective half-time of I-131 in the body and the breast specifically. Regarding radiation exposure in general, a history of frequent diagnostic radiological examinations or accidental irradiation (e.g., in the case of Chernobyl) may play an important role, confounding the interpretation of the impact of RAI.

Nielsen et al. (13) discussed possible confounders influencing breast cancer risk after DTC, including genetic susceptibility, obesity, and hormones (estrogen and thyroid-stimulating hormone). Different histological subtypes of DTC should be studied separately, given findings thus far regarding genetic tumor profiles. Up to now, some studies have been published about obesity as common risk factor for DTC and breast cancer [e.g., (51)]. As to the role of estrogens and thyroid-stimulating hormone as common risk factors for DTC and breast cancer, a number of *in vitro* and animal studies have been published, but only a few studies in humans (52–54). In any case, levothyroxine therapy, aiming at thyroid-stimulating hormone suppression in patients with advanced DTC, is suspected to increase breast cancer risk independent of DTC (55). In addition, endocrine disruptors like nitrate may play a role in the pathogenesis of DTC and breast cancer (56, 57).

While six of nine cohort and case–control studies published from 1984 to 2000 did not show an increased risk for breast cancer after DTC, 15 of 17 studies published in 2000 and later demonstrated such an increased risk. It may be speculated that, in the later period, which is characterized by a sharp increase in DTC incidence worldwide, some common risk factors such as endocrine disruptors may have affected the incidence of breast cancer as well as DTC.

Finally, with respect to the scope of this review, to obtain an estimate of the risk of breast cancer as an SPM after RAI of DTC with a focus on young females, it is difficult to draw proper conclusions. That is because only two studies referenced here concentrated on those given such therapy as children or adolescents (3, 25). These studies described an increasing risk with age after adolescence for different types of SPM after RAI for DTC; however, these tumor entities did not include breast cancer.

Since the data are not sufficient to draw any conclusions about age dependence of breast cancer risk after RAI of DTC, it seems to be reasonable and necessary to evaluate whether relevant data in large-enough samples of young DTC patients can be collected in a systematic international multicenter survey which should be carried out as a case–control study applying multivariate statistical methods.

### International Multi-Registry Survey

Our registry study was performed with the goal of assessing the potential sample size for a larger and more detailed study, allowing firmly evidence-based conclusions to be drawn on the real risk of breast cancer before and after treatment of DTC with RAI in young females. One important finding of our survey was that usually registries covering the whole age spectrum of patients with DTC contain only very low percentages of those <18 years old, ranging between 2 and 7% (the situation in Münster, Würzburg, and Gliwice registries in our survey). Since dedicated registries of children and adolescents with DTC are rare and tend to contain only low numbers of cases, it might not to be possible to restrict the limit of “young age” at the time of RAI to 18 years. To ensure the recruitment of a sample size enabling sufficient statistical power, the age limit at the time of RAI probably should be increased to 40 years, which would cover about 35% of patients with DTC.

Based on the maximum breast cancer rates in our registry survey (see Section Cumulative I-131 Activity) and on published data (5), it is assumed that—independent of treatment—about 2% of female DTC survivors develop breast cancer. As noted earlier, RAI for DTC with cumulative activities of 10–15 GBq I-131 corresponds to radiation doses to the female breast of between 2 and 3 Gy, which may double/triple the lifetime risk for breast cancer (8). We estimated that sample sizes of at least 4,340 cases below age 40 years at the time of RAI and 660 controls will be necessary under the assumption of an HR of 2.76 to reject the zero hypothesis of no effect of RAI, with a power of 80% (58).

Taking into account that about 3,200 of the patients in this registry study already met the age <40 years criterion, it seems to be feasible to recruit 30% more cases by including additional patients from other DTC specialist centers in Germany and abroad. Regarding the controls not receiving RAI, this survey already identified 650 of the 660 necessary patients for a subsequent study.

## Conclusions

To summarize today's state of knowledge, independent of DTC treatment, there appears to be a bi-directional association of DTC itself and breast cancer. Nonetheless, the risk of breast cancer in adult DTC survivors is low, about 2%, slightly higher in females than in males, but based on the literature, presumably lower in children and adolescents than in older age groups. RAI is assumed not to substantially influence the lifetime risk of breast cancer after DTC, but data from those given such treatment as children or adolescents are sparse.

The literature review and multicenter registry survey reported here lead to the following recommendation: an international multicenter study with a sufficiently high number of female DTC survivors is feasible; that study should have a case–control design and include female patients <40 years of age with DTC diagnosed 20–30 years earlier. For reasons of compliance and practicability, such a design should be preferred over a longitudinal study lasting several decades.

However, especially given the likely at most slightly increased risk for breast cancer after RAI, breast cancer screening of a large cohort of female DTC survivors who received breast doses between 0.2 and 2 Gy from cumulative RAI activities of 1–15 GBq I-131 (7) is not unproblematic for ethical reasons. These reasons are related to an expected high rate of false-positive findings of ultrasonographic screening for breast cancer—in the range of 10%—and a resultant uncertain frequency of “misdiagnosis.” On similar grounds, the International Late Effects of Childhood Cancer Guideline Harmonization Group does not recommend routine breast cancer surveillance in female childhood, adolescent, or young adult cancer survivors treated with chest radiation doses <10 Gy (59). Such concerns apply in an even more pronounced way to the control cohort not affected by RAI-related radiation exposure as a potential risk-increasing factor. Hence, RAI patients and controls should be especially actively involved in choices regarding breast cancer screening, in a “shared decision-making” approach, and particular attention should be paid to the education of potential study participants regarding this possible issue (60).

## Author Contributions

CR contributed to concept development, study management, critical review of the literature, and editing of the final manuscript. RS contributed to study management, literature search, critical review of the literature, and language editing. TP contributed to the management and the analysis of thyroid cancer database in Minsk. MF contributed to the histology review of thyroid cancer database in Minsk. UMä contributed to the supply of registry data from Würzburg. UMa contributed to the statistical analysis and writing the statistics section of the manuscript. AV contributed to the supply of registry data from Münster. TB contributed to the supply of registry data from Kiev. JK contributed to the supply of registry data from Gliwice. RE contributed to the supply of registry data from Pisa. FV contributed to the supply of registry data from Rio de Janeiro. JM contributed to the supply of registry data from Sremska Kamenica. GC contributed to the supply of registry data from Coimbra. VD contributed to the study management, literature search, critical review of the literature, supply of feasibility study data from Minsk, and drafting of the manuscript. All authors contributed to the critical review. All authors contributed to the article and approved the submitted version.

## Conflict of Interest

The authors declare that the research was conducted in the absence of any commercial or financial relationships that could be construed as a potential conflict of interest.
